# Proteomic investigation of acute and chronic hypoxia/reoxygenation responsive proteins and pathways in H9C2 cardiomyoblasts

**DOI:** 10.55730/1300-0152.2695

**Published:** 2024-04-24

**Authors:** Merve ÖZTUĞ, Evren KILINÇ, Zeynep A. ÖZTUĞ DURER, Emel BALOĞLU

**Affiliations:** 1 TÜBİTAK National Metrology Institute (TÜBİTAK UME), Kocaeli, Turkiye; 2 Department of Molecular Biology and Genetics, Faculty of Science and Letters, Istanbul Technical University, İstanbul, Turkiye; 3 Department of Biophysics, Hamidiye School of Medicine, University of Health Sciences, İstanbul, Turkiye; 4 Department of Biophysics, School of Medicine, Acıbadem Mehmet Ali Aydınlar University, İstanbul, Turkiye; 5 Department of Biochemistry, Faculty of Pharmacy, Acıbadem Mehmet Ali Aydınlar University, İstanbul, Turkiye; 6 Department of Medical Pharmacology, School of Medicine, Acıbadem Mehmet Ali Aydınlar University, İstanbul, Turkiye; 7 Department of Medical Pharmacology, School of Medicine, İstanbul Medeniyet University, İstanbul, Turkiye

**Keywords:** Ischemic heart disease, H9C2 cardiomyoblast, differential proteomics, acute hypoxia, chronic hypoxia

## Abstract

**Background/aim:**

Ischemic heart diseases continue to be a significant global cardiovascular problem in today’s world. Myocardial reperfusion (R) is provided with an effective and rapid treatment; however, it can lead to fatal results, as well as ischemia (I). This study aims to use proteomic analysis to assess proteins and pathways in H9C2 cardiomyoblast cells exposed to hypoxic conditions, followed by reoxygenation, representing I/R injury for both short and long terms, reflecting acute and chronic hypoxia, respectively. Utilizing advanced techniques, our goal is to identify and characterize key proteins undergoing alterations during these critical phases.

**Materials and methods:**

H9C2 cardiomyoblasts, a commonly used cell line for simulating in vivo I/R damage, were exposed to normoxia and hypoxia (0.4% O_2_) in six experimental groups: normoxia (3h), acute hypoxia (3h), acute hypoxia (3h) + reoxygenation (3h), normoxia (21h), chronic hypoxia (21h), and chronic hypoxia (21h) + reoxygenation (3h). Analyses were conducted using Nano LC/MSMS from tryptic digest of the whole cell lysates. Proteins were quantified using the label-free quantification (LFQ) algorithm in Proteome Discoverer 2.4.

**Results:**

Proteomic analysis resulted in identification of 2383 protein groups. Proteins that differentially expressed in the various groups were identified (p < 0.05 among mean values for groups). Short-term hypoxia induces mitochondrial damage, energy demand, and cytoskeletal modifications. Chronic hypoxia triggers metabolic shifts, stress-response proteins, and extracellular matrix alterations. Data are available via ProteomeXchange with identifier PXD047994.

**Conclusion:**

Our research provides in-depth insights into how H9C2 cardiomyoblasts respond to both short-term and prolonged oxygen deprivation. Understanding hypoxia-related pathophysiology provides avenues for therapeutic intervention in hypoxia-related disorders.

## 1. Introduction

Ischemia, characterized by reduced blood flow, is a fundamental factor in cardiovascular diseases, particularly ischemic heart disease, which is a leading cause of global morbidity and mortality (Shimokawa and Yasuda, 2008). Short-term ischemia occurs when there is a temporary reduction in blood flow, such as during an acute coronary event or heart attack. In these situations, prompt reperfusion therapy is crucial for salvaging the ischemic myocardium. However, the restoration of blood flow, while essential, can paradoxically lead to further damage during reperfusion. The reperfusion phase introduces oxidative stress and triggers a cascade of events, including hypoxia in the affected tissue, which persists for days or weeks. Long-term ischemia, resulting from chronic conditions, exacerbates the injury and contributes to cardiovascular diseases like heart failure, myocardial ischemia, and infarction (Rentrop and Feit, 2015; Naito et al., 2020; Chang et al., 2021).

Cardiomyocytes, the essential contractile cells of the heart, play a pivotal role in the response to ischemic stress and subsequent reperfusion injury. Upon encountering pathological stimuli, such as biomechanical stress induced by hypoxia, cardiomyocytes undergo alterations in their morphology, protein synthesis, and reactivate genes associated with fetal cardiac development (Thum et al., 2007; Boccellino et al., 2018). Unfortunately, these initially adaptive changes can evolve into maladaptive responses, ultimately contributing to adverse ventricular remodeling and heart failure through the atypical activation of signaling pathways. Consequently, understanding the complex cell signaling events that regulate cardiac function becomes crucial to develop well-founded therapeutic strategies for preventing heart diseases (Yung et al., 2004). Previous studies have demonstrated examples of such signaling pathways. For instance, G protein-coupled receptor (GPCR) and tyrosine kinase receptor (RTK), through the activation of ERK1/2, play a potential role in hypertrophic cardiomyopathy treatment (Migliaccio et al., 1991; Castoria et al., 2008; Aquino et al., 2012). Rho and Rac are implicated in signaling pathways that regulate various cellular functions, such as actin stress fiber assembly and focal adhesions in myocardial hypertrophy and heart failure (Buommino et al., 2007; Borghese et al., 2017; Ricci et al., 2019). Alongside RhoA, Rac1 is a well-characterized small G protein in myocardial signaling, regulating hypertrophic remodeling through MAPK cascade activation (Ridley et al., 1992; Brown et al., 2006). Mechanical stretch, reoxygenation damage, and ischemia/reperfusion injury induce ROS formation, linked to Rac1 activation (Acevedo and González-Billault, 2018). Activation of the Rho kinase pathway affects cardiomyocytes, leading to inflammatory and proliferative changes in blood vessels.

H9C2 cardiomyoblasts hold significance in cardiac research owing to their close similarity to normal primary cardiomyocytes in terms of energy metabolism, heightened sensitivity to hypoxia, and their appropriateness for creating in vitro models that mimic cardiac hypoxia-reoxygenation and myocardial infarction (Kuznetsov et al., 2015). Prior studies have explored hypoxia-related protein markers in these cells including proteomics approaches (Li et al., 2014; Ai et al., 2015; Xu et al., 2016; Boccellino et al., 2021; Liu et al., 2023). However, to our knowledge, no quantitative proteomic study encompassing both acute and chronic hypoxia conditions has been conducted previously. Understanding both short and long-term ischemia and reperfusion dynamics is crucial for developing effective therapeutic approaches to mitigate the impact of cardiovascular diseases on a global scale. In this study, we aim to analyze the response of H9C2 cells to hypoxia, employing proteomic analysis to assess alterations in proteins and pathways within H9C2 cardiomyoblast cells exposed to ischemia and reperfusion, mimicking short- and long-term conditions. Employing advanced techniques, we seek to identify and characterize key proteins modulated during these critical phases and investigate cellular responses to hypoxia, including cell proliferation, apoptosis, oxidative stress, and molecular pathways.

## 2. Materials and methods

### 2.1. Chemicals and materials

### 2.2. Cell culture

H9C2 cells (rat ventricular cardiomyoblasts) (ATCC, Manassas, VA, USA) were cultured as described previously (Gurler et al., 2023). The medium was replaced every two days, and cells were grown until confluency reached 80%. For the experiments, cells were seeded at a density of 2 × 10^5^/cm^2^.

### 2.3. In vitro hypoxia/reoxygenation injury

**Figure 1:**
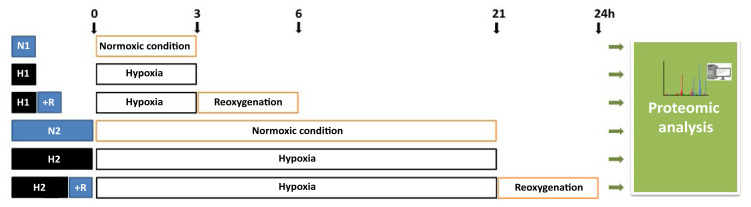
Study design for comparative proteome analysis in H9C2 cardiomyoblasts exposed to acute (N1, H1, H1+R) or chronic (N2, H2, H2+R) hypoxia and reoxygenation injury. **N1:** Normoxic condition for 3 h, **H1:** Hypoxia for 3 h, **H1+R:** Reoxygenation for 3 h after 3 h of hypoxia, **N2:** Normoxic condition for 21 h, **H2:** Hypoxia for 21 h, **H2+R:** Reoxygenation for 3 h after 21 h of hypoxia.

For in vitro hypoxia experiments, cells were kept in an incubator in 0.4% O_2_ + 5% CO_2_, residual N_2_ gas at 37 °C (Binder, Tuttlingen, Germany). Normoxia (acute N1 and chronic N2) or reoxygenation (R) was established at 19% O_2_ and 5% CO_2_. Two different hypoxia regimes were applied and identified as acute and chronic. The experimental design for acute (3h) and chronic (21h) hypoxia with and without reoxygenation (3h) is shown in Figure 1. To generate acute hypoxia and reoxygenation injury, H9C2 cells were subjected to 3 h of hypoxia (0.4% O_2_; H1) or normoxia (19% O_2_ ; N1) and 3 h of reoxygenation followed by hypoxia (H1 +R). In parallel, 21 h of hypoxia (H2) or normoxia (N2) and 3 h of reoxygenation followed by hypoxia (H2 +R) were applied for long-term hypoxia and reoxygenation injury experiments. Subsequently, cells were harvested, lysed, trypsin-digested, and analyzed using a bottom-up label-free proteomic approach, as outlined in Figure 1.

### 2.4. Cell lysis and protein digestion

Cells were washed three times with ice-cold PBS and lysed in a RIPA buffer (Thermoscientific, Germany) composed of 1 mM PMSF, and 1X protease inhibitor cocktail for 20 min at 4 °C and centrifuged at 14,000 × *g* for 10 min at 4 °C. Supernatants containing total cellular proteins were frozen at −80 °C until use. Protein concentration was determined using the Qubit™ Protein Assay kit (Invitrogen, USA). Subsequently, 50 μL of the protein extract underwent enzymatic digestion employing filter aided sample preparation (FASP) with a trypsin enzyme-to-protein ratio of 1:25 using the FASP Protein Digestion Kit (Expedeon Inc., San Diego, CA, USA). The elution was done with 40 μL of 50 mM NH_4_HCO_3_ and 50 μL of 0.5 M sodium chloride. Samples were diluted to a final concentration of 200 ng/μL with 0.1% formic acid before being transferred to HPLC vials.

### 2.5. LC–MS/MS analysis

Analyses were performed in Thermo Scientific Q Exactive HF-X mass spectrometer (MS) coupled with Thermo Scientific UltiMate 3000 RSLC Nano Ultra performance liquid chromatography (UPLC) as described previously (Mumcu et al., 2023). Briefly, peptide separation involved reversed-phase liquid chromatography (RP-LC) using Acclaim PepMap C18 Trap Cartridge (5 μm, 100A, 300 μm i.d × 5 mm) and EASY-Spray ES902 RSLC C18 (2 μm, 100A 75 μm × 25 cm) columns. The column temperature was set at 40 °C with a flow rate of 350 nL/min, and 500 ng of peptides were loaded. Mobile Phase A and Mobile Phase B comprised 0.1% FA, 98:2% H2O: ACN, and 0.1% FA, 98:2% ACN:H2O, respectively. The gradient started at 3%, reaching 80% ACN over 70 min, followed by a 20-min reequilibration with 3% B solution. Instrument parameters included a spray voltage of 2 kV, funnel RF level 50, and a capillary temperature of 270 °C. The device operated in “Full MS/DD–MS/MS” configuration for data-dependent analysis (DDA), with Full MS resolution set at 60,000 for m/z 200. Full MS AGC target was 3E6 at 45 ms, and the mass range was defined as 350–1400. MS/MS AGC target value was 1E5, with a resolution of 15,000, a maximum IT of 22 ms, an intensity threshold of 2E4, and an isolation width of 1.3 m/z. The normalized collision energy was set at 28%, and all data were acquired in positive ion mode. The mass spectrometry proteomics data have been deposited to the ProteomeXchange Consortium via the PRIDE (Vizcaíno et al., 2015) partner repository with the dataset identifier PXD047994 and 10.6019/PXD047994.

### 2.6. Protein identification and quantification

Raw data files were searched against the NCBI Rattus Norvegicus database (Taxonomy ID: 10116) utilizing the Sequest HT search engine (Eng et al., 1994), with a stringent false discovery rate (FDR) value set at 0.01. Sequest HT parameters included a precursor mass tolerance of 10 ppm and a fragment mass tolerance of 0.02 Da. The search parameters specified a fixed modification of cysteine carbamidomethylation and variable modifications of oxidation and protein N-terminal acetylation. Proteins were identified with a minimum of two peptides, each having a length of at least six amino acids. Quantification of proteins was achieved using the label-free quantification (LFQ) algorithm within Proteome Discoverer 2.4. Significant differences in protein abundances, with a greater than 2-fold change (log2 FC: 1), were assessed using Proteome Discoverer false discovery rate-adjusted p-values (p < 0.05).

## 3. Results

### 3.1. LC-MSMS analysis

**Figure 2:**
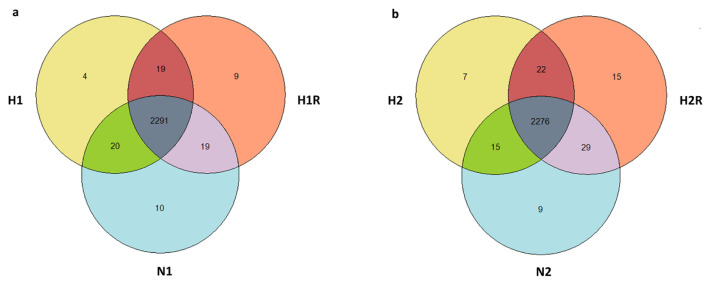
Venn diagrams illustrating protein dynamics under a) acute and b) chronic hypoxic conditions.

Hypoxic stress, characterized by reduced oxygen availability, can have profound effects on cellular function and homeostasis. Inducing hypoxic stress can elicit acute and chronic cellular responses. Understanding the molecular mechanisms underlying these responses is crucial for elucidating the pathophysiology of hypoxia-related conditions and identifying potential therapeutic targets. To create hypoxic conditions, various methods have been developed and employed in different research areas. In this study, cells were cultured in a sealed humidified chamber with 5% CO_2_ and 95% nitrogen to simulate hypoxic stress conditions. The experimental workflow involved subjecting cells to different durations of hypoxia, with or without subsequent reoxygenation, mimicking acute and chronic hypoxic stress scenarios. Following this, cells were collected, lysed, trypsin-digested, and subjected to analysis using a bottom-up label-free proteomic approach. The proteomics results obtained from the comparative LFQ using Nano LC/MSMS led to the identification of 2.383 protein groups, 13.418 peptide groups, and 228,485 PSMs. The differential expression analysis highlighted specific protein dynamics, with 4 proteins exclusively identified under acute hypoxia, 10 proteins unique to normoxia, 9 proteins unique to reoxygenation, and 2291 proteins common to both conditions. These findings were illustrated in a Venn diagram (Figure 2a). Similarly, in the context of chronic hypoxia, the analysis identified 7 proteins exclusively present, 9 proteins unique to normoxia, 15 proteins exclusive to reoxygenation, and 2276 proteins common to both conditions (Figure 2b). This approach yielded statistically significant differences in specific protein profiles among the distinct groups. The complete list of proteins, including normalized abundance values, ratios, and adjusted p-values, can be found in the Supplementary material.

### 3.2. Acute hypoxia/reoxygenation

**Figure 3:**
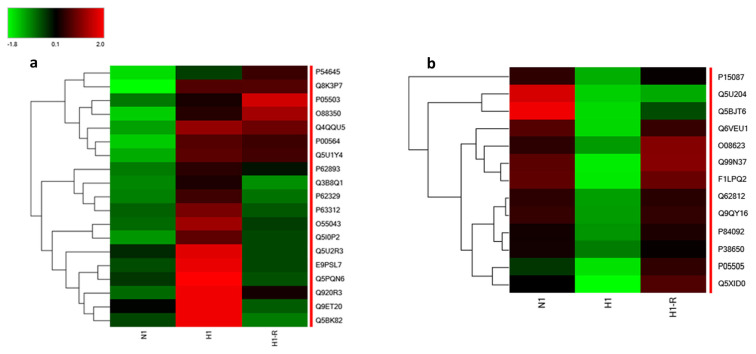
Differential protein expression profiles in response to acute hypoxic conditions. a) Heat map depicting the increased expression levels of 19 proteins during H1, N1, and H1R conditions, b) heat map representing the decreased expression levels of 13 proteins during H1, N1, and H1R conditions.

The proteomic analysis revealed significant alterations in protein expression under acute hypoxic conditions. Specifically, the criteria for inclusion involved a greater than two-fold change in abundance (log2 FC: 1, p < 0.05, and FDR<1.0%). Notably, 19 proteins exhibited substantial upregulation (>2-fold), 13 proteins were significantly downregulated (0.5-fold), and 2211 proteins maintained normal expression levels (0.5–2-fold) under normoxia. The differentially expressed proteins during acute hypoxia conditions are listed in Table 1. The heat maps illustrating the 19 upregulated proteins and the 13 downregulated proteins during H1 compared to N conditions are presented in Figures 3a and 3b, respectively. Importantly, after a 3-h reoxygenation period (H1R), some upregulated protein levels returned to normoxic levels, indicating a reversible response. Similar to the upregulated proteins, it was observed that the levels of most of these downregulated proteins returned to baseline during reoxygenation, highlighting a reversible response to hypoxic conditions.

### 3.3. Chronic hypoxia/reoxygenation

**Figure 4:**
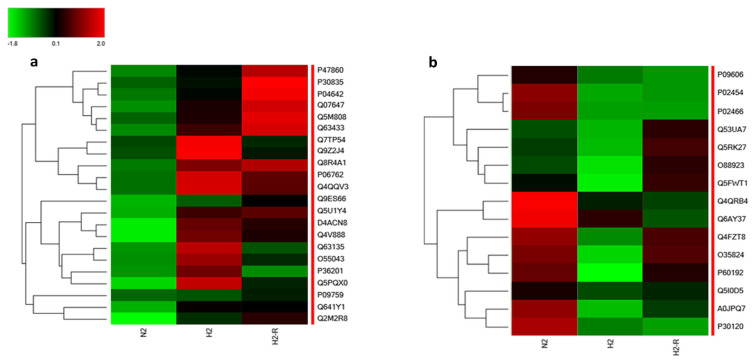
Differential protein expression profiles in response to chronic hypoxic conditions. a) Heat map depicting the increased expression levels of 19 proteins during H2, N2, and H2R conditions, b) heat map representing the decreased expression levels of 13 proteins during during H2, N2, and H2R conditions.

The proteomic analysis revealed significant alterations in protein expression under chronic hypoxic conditions. Specifically, the criteria for selection involved a greater than two-fold change in abundance (log2 FC: 1, p < 0.05, and FDR < 1.0%). Notably, 22 proteins exhibited substantial upregulation (>2-fold), 15 proteins were significantly downregulated (0.5-fold), and 2165 proteins maintained normal expression levels (0.5–2-fold) under normoxia. The differentially expressed proteins during chronic hypoxia conditions are listed in Table 2. The heat maps depicting the 22 upregulated proteins and the 15 downregulated proteins during H2 compared to N2 conditions were presented in Figures 4a and Figure 4b, respectively. Significantly, after a 3-h reoxygenation period (H2R), some upregulated protein levels reverted to normoxic levels, indicating a reversible response. Similar to the upregulated proteins, it was observed that the levels of most of these downregulated proteins returned to baseline during reoxygenation, highlighting a reversible response to hypoxic conditions.

### 3.4. Enrichment analysis

STRING Functional Enrichment Analysis is a powerful bioinformatics tool used to elucidate the biological significance of large sets of proteins by identifying enriched functional annotations, such as pathways, biological processes, and molecular functions (Szklarczyk et al., 2014). In this analysis, we explored the biological interactions and functions of proteins that showed significant alterations. In the acute hypoxia model, specific pathways associated with a notable increase or decrease in protein expression were identified. The results are presented in Table 3. In the context of acute hypoxia, our pathway enrichment analysis identified two significantly enriched reactome pathways. Notably, TP53 regulates metabolic genes (false discovery rate = 0.0077) and macroautophagy (false discovery rate = 0.0234) emerged as key pathways responding to short-term hypoxic conditions.

Similarly, in the chronic hypoxia model, the biological contexts of the differentially expressed protein groups were examined. The results are also presented in Table 3. Conversely, in chronic hypoxia, a more diverse range of enriched pathways emerged across different databases. Notably, the KEGG pathway analysis revealed significant enrichment in the HIF-1 signaling pathway (false discovery rate = 0.00037), indicating the activation of hypoxia-inducible factor 1. Additionally, glycolysis/gluconeogenesis, central carbon metabolism in cancer, biosynthesis of amino acids, and the glucagon signaling pathway were also enriched, reflecting the multifaceted adaptations occurring in cells subjected to prolonged hypoxia.

Moreover, reactome pathway analysis showed enrichment in the degradation of the extracellular matrix, suggesting potential alterations in tissue structure during chronic hypoxia. In Wiki pathways, insights were gained into diverse processes such as fructose metabolism in proximal tubules, inflammatory response pathway, cardiovascular signaling, and hexoses metabolism in proximal tubules. These findings underscore the complexity of cellular responses during prolonged hypoxia, involving diverse molecular pathways that extend beyond the acute phase.

## 4. Discussion

Hypoxia, a common condition observed in ischemia, may trigger different signaling pathways depending on the duration and severity of the low oxygen stress. While acute and short-term hypoxic stress is associated with myocardial infarction; prolonged hypoxia or chronic hypoxia refers to cardiorespiratory disease including heart failure. Short-term hypoxia mainly shuts down the ATP consuming processes, while long-term hypoxia turns on and off transcription and translation of some genes controlled by hypoxia-inducible transcription factors (HIFs) to allow adaptation of the cells to low oxygen tension for survival. Our results revealed differential changes in the proteins cells exposed to acute and chronic hypoxia compared to their respective normoxic controls. Remarkably, in short-term hypoxia, upregulation of proteins associated with mitochondrial function, such as cytochrome c oxidase subunit 1, suggests increased mitochondrial damage. Increased AMPK expression indicates increased cellular energy demand for maintaining homeostasis when cellular ATP levels declined (Bairwa et al., 2016; Dengler, 2020) by hypoxia, which is HIF-1a-dependent. In short-term hypoxia, we also observed increased expression of E3 ubiquitin-protein ligase TRIM69, which is known to increase in myocardial infarction and is most likely involved in protein homeostasis (Borlepawar et al., 2019). Moreover, the observed changes in cytoskeletal and cellular structure proteins, including Thymosin beta-4, Thymosin beta-10, and Rho guanine nucleotide exchange factor 7, point to potential modifications in cytoskeletal dynamics and cell motility, reflecting a swift cellular response to acute hypoxia. Conversely, proteins associated with cytoskeletal components, such as Cytoplasmic dynein 1 heavy chain 1 and Myosin-9, show decreased expression, indicating potential changes in cytoskeletal organization during acute hypoxia. Additionally, alterations in cellular processes and signaling proteins, including Rho GTPase-activating protein 17 and RNA-binding protein NOB1, suggest dynamic shifts in cellular signaling and protein regulation in response to short-term hypoxic stress. The subtle alterations in protein expression observed during acute hypoxia highlight cellular adaptations crucial for immediate survival when dealing with fluctuations in oxygen levels.

In chronic hypoxia, our study uncovered more detailed insights into how cells respond to long-term low oxygen levels. Lactate dehydrogenase, a HIF-dependent gene that converts pyruvate to lactate, was increased in long-term hypoxia. Notably, the increased expression of L-lactate dehydrogenase A chain and ATP-dependent 6-phosphofructokinase, liver type, indicates adaptations in energy metabolism, emphasizing the cell’s efforts to cope with sustained hypoxic conditions. These changes underscore a metabolic shift, likely aimed at optimizing energy production pathways for prolonged survival under chronic hypoxia.

In addition, in response to both short-term and long-term hypoxic conditions, a 3-h reoxygenation period generally led to the restoration of most proteins to normoxic levels, indicating a reversible adaptation to hypoxia. However, a subset of proteins did not revert to normoxic levels during reoxygenation. These proteins that persistently remain at altered levels during reoxygenation may play a crucial role in the damage associated with reperfusion, reflecting ischemia and reperfusion injury. Since myocardial reperfusion can have fatal consequences, understanding the proteins that do not return to baseline levels after reoxygenation becomes crucial for developing therapeutic strategies to mitigate reperfusion injury. Identifying and targeting these specific proteins could be a promising approach in therapeutics. These proteins may represent key players in the cellular response to reperfusion, and interventions aimed at modulating their activity or expression could potentially reduce the damage caused during reoxygenation.

Furthermore, our findings highlight cellular stress and adaptation responses during chronic hypoxia. The upregulation of heme oxygenase 1 and ERO1-like protein alpha suggests active responses to oxidative stress and cellular adaptation strategies employed by cells enduring prolonged oxygen deprivation. The increased degradation of heme proteins by heme oxygenase 1, which necessitates heme synthesis for biochemical processes, is a feature of chronic hypoxia (Neubauer and Sunderram, 2012). The increased expression of peroxisomal targeting signal-1 which directs the proteins into peroxisomes (Gatto et al., 2000) might indicate that under hypoxic conditions peroxisome homeostasis required adaptations. Conversely, proteins associated with extracellular matrix and cellular structure exhibit decreased expression in chronic hypoxia. Collagen alpha-1 (I) chain, collagen alpha-2 (I) chain, and tubulin beta-3 chain show reduced levels, indicating potential modifications in the extracellular matrix and cellular structure during prolonged oxygen deprivation. Moreover, alterations in cellular signaling and regulation proteins, including serine/threonine-protein kinase TAO3, SPRY domain containing protein 4, and metalloproteinase inhibitor 1, further emphasize the intricate adjustments in cellular signaling and regulatory pathways during chronic hypoxia. Together, these changes in how proteins are expressed during extended hypoxia provide insights into the intricate adjustments cells make to survive in prolonged low-oxygen environments.

The results of the enrichment analysis showed that pathways involved in controlling cellular energy homeostasis, amino acid synthesis, and quality control pathways are time-dependently sensitive to hypoxic stress and at first energy demand is warranted. During prolonged hypoxia other cellular processes such as maintaining iron-dependent biochemical reactions and protein quality control related pathways are regulated. The upregulation of “TP53 Regulates Metabolic Genes” and “Macroautophagy” reactome pathways in the context of acute hypoxia is consistent with the known role of p53 in metabolic regulation and its involvement in autophagy. Research has demonstrated the involvement of p53 in the regulation of metabolic homeostasis, including the control of energy metabolism and reactive oxygen species production (Berkers et al., 2013). Moreover, p53 has been shown to regulate cellular energy metabolism and influence mitochondrial energy production, impacting ATP levels (Liu et al., 2015).

KEGG pathways in chronic hypoxia, including “glycolysis/gluconeogenesis,” “central carbon metabolism,” and “hypoxia-inducible factor 1 (HIF-1) signaling pathway,” suggests a metabolic reprogramming in response to chronic hypoxia. This is consistent with the known role of HIF-1 in regulating cellular metabolism and promoting glycolysis under hypoxic conditions (Semenza, 2009). Additionally, the enrichment of the “HIF-1 signaling pathway” which plays a central role in the cellular response to low oxygen levels further supports the involvement of HIF-1 in orchestrating adaptive responses to chronic hypoxia, including angiogenesis and metabolic reprogramming (Semenza, 2009). The enrichment of “biosynthesis of amino acids” pathway indicates potential alterations in amino acid metabolism in response to chronic hypoxia. This is in line with the cellular adaptations to hypoxia, where amino acid metabolism plays a crucial role in maintaining cellular homeostasis under low oxygen conditions (Song et al., 2020). Furthermore, the enrichment of the “glucagon signaling pathway” suggests a potential role for glucagon in modulating cellular responses to chronic hypoxia. Glucagon has been implicated in regulating glucose metabolism and insulin signaling, and its involvement in the context of chronic hypoxia may reflect the complex interplay between hormonal regulation and metabolic adaptations (Li et al., 2019). Degradation of the extracellular matrix indicates potential remodeling of the extracellular matrix in response to chronic hypoxia. In summary, these pathways reflect multifaceted cellular responses to prolonged oxygen deprivation.

Overall, the application of functional enrichment analysis in the context of acute and chronic hypoxia models allowed for a systematic exploration of the biological interactions and functions of proteins that exhibit significant alterations in expression. This approach not only enhances our understanding of hypoxia-related pathophysiology but also offers potential targets for therapeutic intervention in hypoxia-related disorders.

In conclusion, our comprehensive analysis of protein expression patterns in response to acute and chronic hypoxia has provided valuable insights into the dynamic cellular adaptations to varying durations of low oxygen stress. Acute hypoxia induces rapid changes in energy regulation, evidenced by the upregulation of AMPK and proteins associated with cellular energy production. Concurrently, alterations in cytoskeletal dynamics and cellular motility suggest a quick cellular response to oxygen fluctuations. On the other hand, chronic hypoxia prompts sustained adaptations, including metabolic reprogramming, cellular stress responses, and structural changes, as reflected in the differential expression of proteins involved in energy metabolism, oxidative stress response, glucose transport, and extracellular matrix remodeling. By carefully examining the molecular responses within these distinct timeframes, our research gives us a detailed understanding of how hypoxia affects cells over time. This knowledge is crucial for developing targeted therapeutic treatments that consider the specific challenges posed by acute and chronic ischemic conditions. As we keep investigating how cells respond during oxygen loss and recovery, these discoveries help build the basis for treatments aimed at reducing heart damage in various ischemic situations.

## Supplementary Information



## Figures and Tables

**Table 1:** Differentially expressed proteins in acute hypoxia conditions.

Accession	Description	p-values
**Acute increased**		
P54645	5′-AMP-activated protein kinase catalytic subunit alpha-1	0.000232725
P62893	60S ribosomal protein L39	1.53331E-14
Q5U1Y4	1,5-anhydro-D-fructose reductase	3.53047E-12
P62329	Thymosin beta-4	0.001592435
P63312	Thymosin beta-10	0.008388377
O88350	Serine hydrolase RBBP9	9.17875E-05
Q5I0P2	Glycine cleavage system H protein, mitochondrial	0.04104725
Q9ET20	Secretory carrier-associated membrane protein 4	0.002330713
O55043	Rho guanine nucleotide exchange factor 7	6.89291E-06
P00564	Creatine kinase M-type	0.001536233
Q4QQU5	Protein YIPF6	0.009144198
Q920R3	Acyl-CoA (8-3)-desaturase	1.04642E-08
Q3B8Q1	Nucleolar RNA helicase 2	0.005656053
Q5PQN6	Spermatogenesis-defective protein 39 homolog	0.005507263
Q8K3P7	Adenosine 5′-monophosphoramidase HINT3	0.014049086
E9PSL7	Citron rho-interacting kinase	7.00309E-05
Q5U2R3	FERM domain-containing protein 8	9.72919E-05
P05503	Cytochrome c oxidase subunit 1	0.033858741
Q5BK82	E3 ubiquitin-protein ligase TRIM69	6.93452E-05
**Acute decreased**		
P38650	Cytoplasmic dynein 1 heavy chain 1	0.001981488
Q62812	Myosin-9	0.005507263
P84092	AP-2 complex subunit mu	0.012658502
P15087	Carboxypeptidase E	1.42753E-10
F1LPQ2	Activating signal cointegrator 1 complex subunit 3	8.45946E-10
O08623	Sequestosome-1	3.24489E-05
Q99N37	Rho GTPase-activating protein 17	1.67603E-05
Q6VEU1	RNA-binding protein NOB1	0.012642975
Q5XID0	Protein YIPF5	1.31425E-07
Q5U204	Regulator complex protein LAMTOR3	0.001753692
Q5BJT6	Large subunit GTPase 1 homolog	0.027761271
P05505	Cytochrome c oxidase subunit 3	3.36668E-05
Q9QY16	ATP-dependent RNA helicase DDX25	0.028526302

**Table 2:** Differentially expressed proteins in chronic hypoxia conditions.

Accession	Description	p-values
**Chronic increased**		
P04642	L-lactate dehydrogenase A chain	1.32235E-05
P30835	ATP-dependent 6-phosphofructokinase, liver type	8.05085E-08
P06762	Heme oxygenase 1	1.56564E-16
P47860	ATP-dependent 6-phosphofructokinase, platelet type	3.39316E-15
Q8R4A1	ERO1-like protein alpha	1.56564E-16
O55043	Rho guanine nucleotide exchange factor 7	1.69661E-06
Q63433	Serine/threonine-protein kinase N1	8.76119E-12
Q07647	Solute carrier family 2, facilitated glucose transporter member 3	6.61577E-08
Q2M2R8	Peroxisomal targeting signal 1 receptor	4.86371E-05
Q9Z2J4	Nexilin	1.56564E-16
Q7TP54	Rho family-interacting cell polarization regulator 2	1.1613E-06
D4ACN8	Plasminogen receptor (KT)	1.67407E-06
Q4QQV3	Protein FAM162A	1.56564E-16
Q5U1Y4	1,5-anhydro-D-fructose reductase	1.56564E-16
Q63135	Complement component receptor 1-like protein	1.03506E-06
Q5M808	Protein Hikeshi	0.029882805
Q5PQX0	UDP-glucuronic acid decarboxylase 1	2.96014E-06
Q9ES66	Calpain-10	0.009777426
Q641Y1	Transmembrane reductase CYB561D2	0.001315411
Q4V888	Type 2 phosphatidylinositol 4,5-bisphosphate 4-phosphatase	0.001359204
P36201	Cysteine-rich protein 2	2.78761E-05
P09759	Ephrin type-B receptor 1	6.70882E-05
**Chronic decreased**		
P02454	Collagen alpha-1 (I) chain	4.23611E-10
P02466	Collagen alpha-2 (I) chain	2.48332E-08
Q4QRB4	Tubulin beta-3 chain	0.000495395
Q5FWT1	Protein FAM98A	1.40559E-06
Q5RK27	Solute carrier family 12 member 7	0.027116389
O88923	Adhesion G protein-coupled receptor L2	1.56564E-16
Q53UA7	Serine/threonine-protein kinase TAO3	0.021633618
Q6AY37	Melanoma-associated antigen B16	0.01109568
P09606	Glutamine synthetase	0.000365719
O35824	DnaJ homolog subfamily A member 2	0.001686822
Q4FZT8	SPRY domain-containing protein 4	0.000233167
Q5I0D5	Phospholysine phosphohistidine inorganic pyrophosphate phosphatase	4.35299E-11
P30120	Metalloproteinase inhibitor 1	0.003393746
P60192	SNARE-associated protein Snapin	0.002712843
A0JPQ7	Uncharacterized protein C19orf47 homolog	1.32617E-09

**Table 3:** Results of pathway enrichment analysis.

**Pathway enrichment analysis for acute hypoxi**a		
**reactome pathways**		
***Pathway***	***Description***	***False discovery rate***
RNO-5628897	TP53 regulates metabolic genes	0.0077
RNO-1632852	Macroautophagy	0.0234
**Pathway enrichment analysis for chronic hypoxia**		
**KEGG pathways**		
***Pathway***	***Description***	***False discovery rate***
rno00010	Glycolysis/gluconeogenesis	0.0236
rno05230	Central carbon metabolism in cancer	0.0236
rno04066	HIF-1 signaling pathway	0.00037
rno01230	Biosynthesis of amino acids	0.0254
rno04922	Glucagon signaling pathway	0.0328
**Reactome pathways**		
***Pathway***	***Description***	***False discovery rate***
RNO-1474228	Degradation of the extracellular matrix	0.0420
**Wiki pathways**		
***Pathway***	***Description***	***False discovery rate***
WP3894	Fructose metabolism in proximal tubules	0.0315
WP40	Inflammatory response pathway	0.0330
WP 590	Cardiovascular signaling	0.0432
WP3916	Hexoses metabolism in proximal tubules	0.0118
